# Cosmetic Analysis Using Matrix-Assisted Laser Desorption/Ionization Mass Spectrometry Imaging (MALDI-MSI)

**DOI:** 10.3390/ma6031000

**Published:** 2013-03-13

**Authors:** Diogo Noin de Oliveira, Sabrina de Bona Sartor, Mônica Siqueira Ferreira, Rodrigo Ramos Catharino

**Affiliations:** INNOVARE Biomarkers Lab, Medicine and Experimental Surgery Department, School of Medical Sciences, University of Campinas, Campinas 13083-877, Brazil; E-Mails: diogo1986@gmail.com (D.N.O.); sabrinabsartor@gmail.com (S.B.S.); monicasiq@gmail.com (M.S.F.)

**Keywords:** cosmetics, cosmetomics, analysis, mass spectrometry

## Abstract

A new “omic” platform—Cosmetomics—that proves to be extremely simple and effective in terms of sample preparation and readiness for data acquisition/interpretation is presented. This novel approach employing Matrix-Assisted Laser Desorption/Ionization Mass Spectrometry Imaging (MALDI-MSI) for cosmetic analysis has proven to readily identify and quantify compounds of interest. It also allows full control of all the production phases, as well as of the final product, by integration of both analytical and statistical data. This work has focused on products of daily use, namely nail polish, lipsticks and eyeliners of multiple brands sold in the worldwide market.

## 1. Introduction

The cosmetic industry is one of the most financially promising branches in the pharmaceutical business, as it shows an increasingly effective potential of sales worldwide [1,2,3]. With such an important position, the quality and safety of its products must be observed and good manufacturing protocols must be followed at all times [4]. 

Traditionally, the study and evaluation of chemical compounds in cosmetics is given by the association between a chromatographic technique—such as HPLC or GC—coupled with electrospray ionization (ESI)-Tandem Mass Spectrometry [5]. It is known that cosmetic products are complex mixtures and thus a previous treatment (e.g., extraction and derivatization) should be employed before the analysis itself. However, some of these sample preparation steps may generate a great number of potentially toxic residues, which is not something compatible with the new trends in eco-friendly and customer safety policies. Moreover, with regard to the large-scale analysis scenario in which industry fits, these conventional procedures are somewhat difficult to automate; therefore they become extremely time-consuming [6].

Mass Spectrometry Imaging is a relatively simple and multifunctional technique that was developed in order to identify the spatial distribution (2-dimensional and sometimes 3-dimensional) of compounds in any physical sample, e.g., a tissue section [7] or a drug tablet [8]. This powerful and versatile technique may be adapted to several different types of ionization, such as SIMS (Secondary Ion Mass Spectrometry) [9,10], DESI (Desorption Electrospray Ionization) [11] and the most common and well-developed variation, MALDI [12]. In every case, despite being a simple task, sample preparation must always be the critical and best-established part of the analysis, for it determines the success or failure of the experiment. On a tissue sample, for instance, every detail, from thickness to type of preservation and matrix application, must be taken into account, as the results may vary when a different method is employed [13]. 

The concept of MALDI-MSI is to transform the sample, e.g., a tissue section, into a two-dimensional picture, providing compound dispersion information on each particular area. The operating principle is very straightforward: a laser beam is shot throughout the sample extension and every shot produces a mass spectrum of that particular spot, which in terms of results may be understood as a “pixel” that forms the final chemical image [12]. 

When it comes to cosmetics, no previous work had been found in the literature, as at first glance it may not seem there is likely to be a great interest in this technique for this kind of analysis. Most daily-use cosmetics, make-up for instance, are very basic and relatively cheap preparations, which is the reason why they are so highly disseminated throughout the world [1]. It is not a rare thing to find some premium brands being imitated by lower-budget companies that promote falsification and use prohibited or health-hazardous compounds [14].

Although much has been investigated on heavy-metals and even lipid profiles in lipsticks [15,16,17], no previous work has explored the capability of finding biomarkers that differentiate, for example, new and decomposed/oxidized products. As for nail polishes and eyeliners, no research of either has been made in the fields of pigments and lipid profiling, respectively. 

We now describe a fast and robust technique to evaluate and quantify compounds of interest in common cosmetic matrixes, such as nail polishes, lipsticks and eyeliners using Matrix-Assisted Laser Desorpiton/Ionization Mass Spectrometry Imaging (MALDI-MSI). With virtually no complex sample preparation, this new field, “Cosmetomics”, has been developed as a very interesting and straightforward alternative for both industrial and academic analysis using the principles of MALDI-MSI for product analysis purposes. For lipsticks and eyeliners, it was possible to differentiate: new, in use and expired products based on their lipid profile. For the nail polishes it was possible to quantify the dye, Sudan III (*m/z* 351, [M − H]^−^), which is a potential class three carcinogen [18].

## 2. Results and Discussion

### 2.1. Chemometric Analysis 

**Figure 1:**
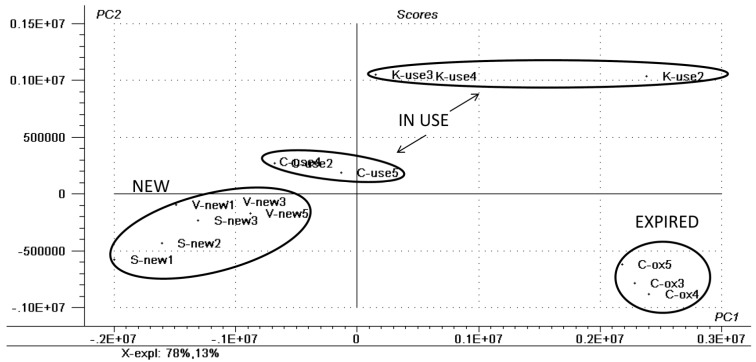
Principal Component Analysis (PCA) score plots showing the different clusters of samples. The three classes (new, in use and expired) are stated.

Principal Component Analysis (PCA) was performed based on characteristic signals observed on each specific sample group. Lipsticks often present a formulation that is rich in very complex lipidic matrixes (up to ~90%), such as *Ricinus communis* seed oil (castor oil), beeswax, Candelilla wax, Carnauba wax, lanolin, *etc.* These matrixes are comprised of a wide range of lipid classes, generally triacylglycerols (TAGs), sphingolipids (SP), free fatty acids and esters, among many others. This great variability in the composition is what gives the characteristic smoothness, thickness, creaminess and even the thixotropic effect of the product. When analyzing and comparing different groups of lipsticks (new, in use and expired) we observed that the lipid composition differs, especially in terms of the oxidation of TAGs and the presence or absence of higher-complexity lipids, such as ceramides. In Figure 1 it is possible to observe the differentiation by PCA between the new, in use and expired lipsticks of multiple brands. 

**Figure 2:**
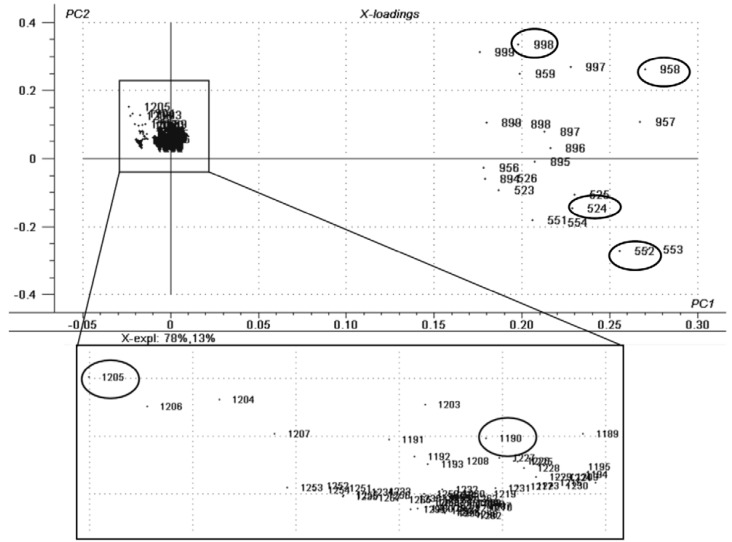
Correlation loadings plot for the principal components in lipstick samples: *m/z* range of 520–560 (diacylglicerols (DAGs)) for the expired product, *m/z* range of 890–1000 (higher triacylglycerols (TAGs)) for the “in-use” products and *m/z* range of 1100–1210 (ceramides) for the new products. The marked ions were the ones chosen as biomarkers for each degradation phase.

This difference is given by the presence of cleaved species in the expired as diacylglicerols (DAGs) that usually lie on the *m/z* range of 500 to 600. The “in-use” samples presented several types of TAGs, whereas the new samples presented sphingolipids (ceramides) as the main difference between the others, as may be seen in Figure 2.

**Figure 3:**
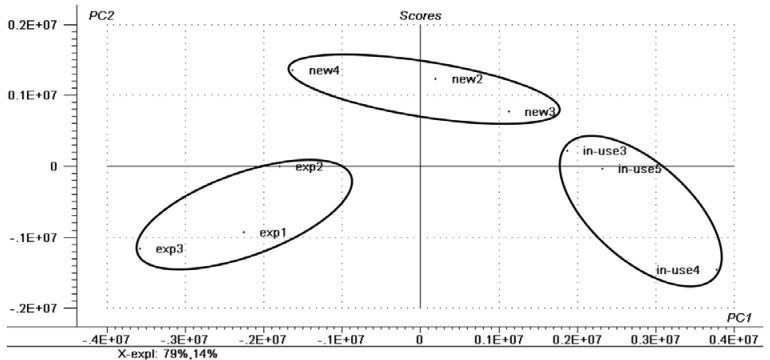
Score plots of the eyeliner sample groups. The groups are represented as “exp” for expired samples, “new” for new samples and “in-use” for used samples.

**Figure 4:**
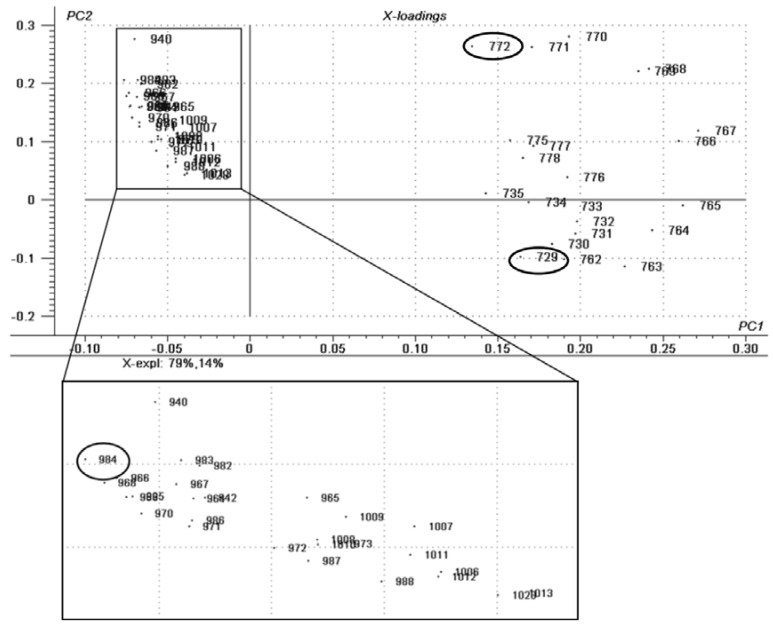
Correlation loadings for the principal components of the eyeliner samples: It is noted there is a very good correlation for both the new and “in-use” groups, as they presented ions in the *m/z* range of 700–800 (lower TAGs). It is possible to observe several ions on the *m/z* 900 and higher. The marked ions were the ones chosen as biomarkers for each degradation phase.

The same principles applied to the eyeliners. The only remarkable difference observed was the presence of oxidized TAGs in the expired product, and because of the insertion of oxygen species in the molecules (epoxy, keto and hydroxyacids), the range of *m/z* increases (~900 and higher). The ions in the mass range from 700 to 800 demonstrate the presence of TAGs both in the new and in the “in use” samples. Figure 3 demonstrates the score plots and Figure 4 the correlation loadings for the different types of samples analyzed. Table I shows the assignment of the characteristic ions detected in each sample group in the positive mode as [M + Na]^+^ species.

The difference between the used and expired samples of eyeliners and lipsticks as to their compositions may be due to the fact that lipsticks are usually in contact with saliva and other compounds around the mouth area; hence, other chemical transformations are more likely to occur than oxidation alone, than with the case of eyeliners.

Table 1 reports the probable assignment for some of the characteristic ions of each sample class. This is based on the MS/MS experiments spectra obtained and their comparison to lipid databases and characteristic fragmentations predicted from the Mass Frontier software. No structural proposal was made, since these *m/z* ratios cannot be assigned to a single structure, as some of them show position isomers within the same lipid class.

**Table 1:** Assignment for characteristic ions [M + Na]^+^ of each sample class.

SAMPLE	LIPID CLASS	CN:DB ^a^	[M + Na]^+^	CID Fragments *m/z*
			*m/z*	
LIPSTICK—NEW	Ceramides	38:1	1190	1146, 1045, 1001
	Ceramides	36:1	1205	1161, 1060, 907
LIPSTICK—USED	Triacylglycerols	58:6	958	914, 844, 660
	Triacylglycerols	60:0	998	700, 419, 363
LIPSTICK—EXPIRED	Diacylglycerols	28:6	524	480, 299, 270
	Diacylglycerols	30:6	552	508, 299, 407
EYELINER—NEW	Triacylglycerols	44:1	772	728, 627
EYELINER—USED	Triacylglycerols	41:2	729	685, 611, 567
EYELINER—EXPIRED	Triacylglycerols	59:0	984	966, 940, 839

^a^ refers to the proposal of carbon number and double bonds number on each molecule.

### 2.2. Quantification by Imaging

The described technique for quantification by image is based on a recent approach utilizing ImageJ [19]. This software is already commonly used as a tool for western blot quantification [20]. The results obtained in this experiment demonstrated that this approach proves to be a useful, rapid and facile tool for semi-quantitative analysis. 

Sudan III was chosen because of its potential carcinogenic risk and because it is also a common dye for cosmetics [21]; in this case, it was stated as an ingredient on all the labels as CI 21600 (color index) and/or Solvent Red 23 (commercial name). The health risks associated with a possible carcinogen in a nail polisher formulation are due to accidental ingestion by nail biting or even when cooking or baking. 

The obtained results are rather interesting and coherent, as the quantity of Sudan III (*m/*z 351, [M − H]^−^) on each sample is directly influenced by the color of the nail polisher. As seen in Table 2, the analyzed products were of different colors, but all of them were listed as “CI 21600” on their labels. Sample CCR, which presented the highest relative concentration, is light blue. It is known that Sudam III is a red-to-brown dye under normal conditions, but when submitted to acidic conditions, turns blue. This particular dye also helps to give a thick and glossy aspect, which was to be expected from sample CCR. The other two samples of highest content (RCR and ICR) are red-to-brown in color. 

**Figure 5:**
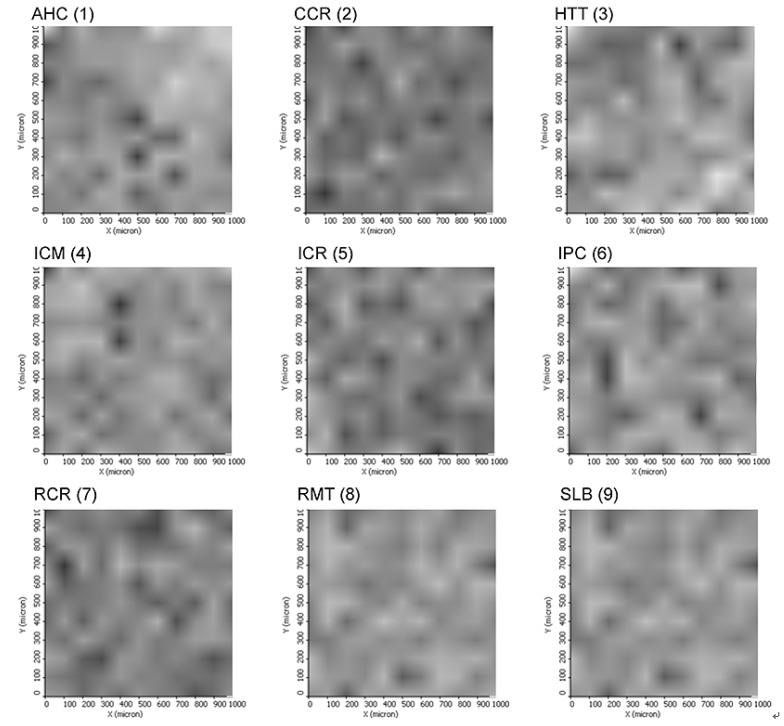
Mass Spectrometry Imaging (MSI) of the samples analyzed on ImageJ software for the semi-quantification. The intensity on the grayscale indicates more (darker) or less (lighter) concentration of the desired analyte. This figure also illustrates that all samples were assigned the same area for further quantitative comparison.

Figure 5 presents the comparison of the different samples (numbered from 1 to 9) in the data table in a graph of intensity on the grayscale.

**Table 2:** Nail polish samples and their color assignment. The third column refers to the observed aspect of the product.

Sample	Color	Aspect
AHC	Dark Blue	Creamy
CCR	Light Blue	Creamy-Glossy
HTT	Brown	Plain
ICM	Red	Metallic
ICR	Orange-Golden	Creamy
IPC	Orange	Plain
RCR	Red	Creamy
RMT	Pink	Metallic
SLB	Golden	Plain

**Figure 6:**
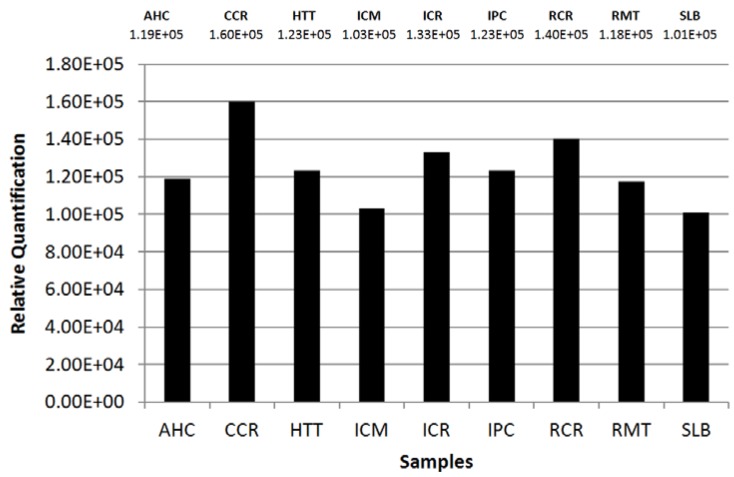
Semi-quantification results. The graph presents a comparison between all the nine samples as to the content of Sudan III (*m/*z 351, [M − H]^−^). It is noticeable that sample CCR has the greatest relative quantity when compared to all the others. The values stated on the top refer to the mean found of the calculations in triplicate. The values are expressed in arbitrary units.

Figure 6 presents the obtained values of the semi-quantification and their comparison in a bar graph. The numbers have no unit scale but represent the relationship between the grayscale intensity and the analyzed area.

**Figure 7:**
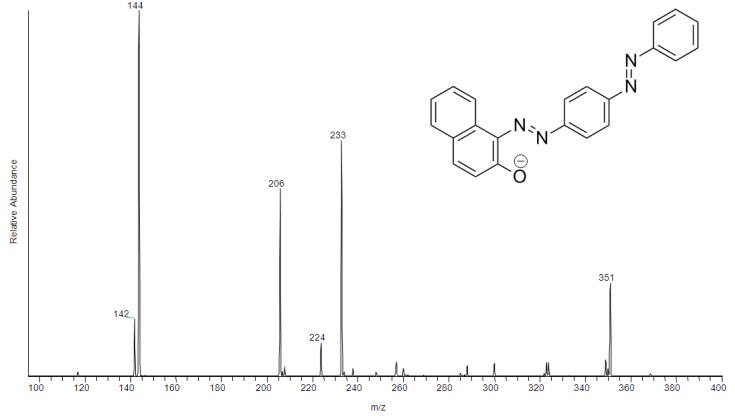
MS/MS spectrum of ion on *m/z* 351—[M − H]^−^ identified as Sudan III.

As seen on Figure 7, the MS/MS spectrum of the ion on *m/z* 351 was identified as Sudan III.

## 3. Experimental Section

### 3.1. Sample Preparation

All cosmetics samples were available and purchased from regular market stores. Nine different samples of nail polishes from six different manufacturers were purchased, all of them indicating Sudan III on the composition (label); for the eyeliners, three different samples (new, used and expired), all from different manufacturers, were purchased; for the lipsticks, five different samples (2 new, 2 used and 1 expired), all from different manufacturers, were purchased. For lipstick application, a soft rod covered in nitrylic polymer was coated with the samples and then “stamped” onto MALDI-appropriate stainless steel plates (GMS-Thermo, California, USA). The eyeliners and nail polishes were directly applied onto the plate surface. A 10 mg/mL solution of alpha-cyano-4-hydroxycinnamic acid (CHCA) matrix (Sigma Aldrich, Pennsylvania, USA) was prepared (50% anetonitrile:methanol) and the samples were coated using a commercially available airbrush. 

### 3.2. MALDI Mass Spectrometry Imaging 

A MALDI-LTQ-XL with Imaging feature (Thermo Fisher, California, USA) was used to acquire mass spectrometric data. Typical operation conditions were 4 µJ Nitrogen laser power, 100 µm raster step size, sample size of 1000 × 1000 µm (100 pixels), three shots per step and 30–50 eV of collision-induced dissociation (CID) for MS/MS experiments. Full scan analyses were performed in a *m/z* range of 50 to 2000. All samples were acquired in the positive ion mode for eyeliners and lipsticks and negative ion mode for the nail polish. The compound classes were proposed both by MS/MS spectra and software calculations with Mass Frontier (v. 6.0, Thermo Scientific, California, USA).

### 3.3. Quantification by Mass Spectrometry Imaging (MSI) 

Imaging data were analyzed in triplicate using ImageQuest software (Thermo Scientific, California, USA). The quantification was performed using ImageJ (National Institutes of Health, USA—Open Source) on grayscale images. The area was standardized in number of pixels for all the replicates and the ImageJ software assigned a value for the selection based on the intensity of each pixel. 

### 3.4. Statistical Analysis of Data 

Principal component analysis (PCA) was performed using Statistica v. 7 (Statsoft Inc., Oklahoma, USA). The mass spectra were expressed as the intensities of individual [M + Na]^+^ ions (*i.e.*, variables). Ions with relative intensities of less than ~10% were excluded. The data was preprocessed using auto scale and the PCA method was run.

## 4. Conclusions 

The development of a new “omic” strategy to employ on the analysis of cosmetics has been successful in the extent of this work. The scope of this new analytical platform is based on manufactured products, considered from raw materials to the final product. Stability studies, degradation byproduct analysis and efficiency of processes are also very feasible to measure and understand with the cosmetomics platform. Viewing the processes and integrating them as a whole—from chemical composition to quantification simultaneously combined with statistical analysis—is, beyond question, the strongest feature of cosmetomics

This novel approach on product analysis will surely help to improve the speed of analysis to a level that the cosmetic industry desires. The possibility of establishing a quick and assertive procedure that can be implemented in the cosmetic industry to perform “right-on-time” analysis (up to 60 s per sample) is very appealing, as many normal analytical procedures are very time-consuming and demand highly-qualified personnel regarding sample preparation, for instance. Requiring little or no sample preparation, not to mention the usage of only very small quantities of solvents and toxic products for analysis, reveals a stark contrast to these other strategies. 

Finally, when it comes to effectiveness and reliability, this new “omic” platform enables the industry to launch products of higher quality and safety for the public. For this reason, several other studies will undoubtedly follow this trend and new applications will be forthcoming.
